# Affective Computing Based on Morphological Features of Photoplethysmography for Patients with Hypertension

**DOI:** 10.3390/s22228771

**Published:** 2022-11-13

**Authors:** Sung-Nien Yu, I-Mei Lin, San-Yu Wang, Yi-Cheng Hou, Sheng-Po Yao, Chun-Ying Lee, Chai-Jan Chang, Chih-Sheng Chu, Tsung-Hsien Lin

**Affiliations:** 1 Department of Electrical Engineering, National Chung Cheng University, Chiayi 621301, Taiwan; 2 Pervasive Artificial Intelligence Research (PAIR) Labs, Hsinchu 300093, Taiwan; 3 Department of Psychology, College of Humanities and Social Sciences, Kaohsiung Medical University, Kaohsiung 80708, Taiwan; 4 Department of Medical Research, Kaohsiung Medical University Hospital, Kaohsiung 80708, Taiwan; 5 Division of Family Medicine, Kaohsiung Medical University Hospital, Kaohsiung 80708, Taiwan; 6 Departments of Family Medicine, Kaohsiung Medical University, Kaohsiung 80708, Taiwan; 7 Division of Cardiology, Department of Internal Medicine, Kaohsiung Medical University Hospital, Kaohsiung 80708, Taiwan; 8 Department of Internal Medicine, College of Medicine, Kaohsiung Medical University, Kaohsiung 80708, Taiwan

**Keywords:** photoplethysmography (PPG), artificial intelligence (AI), affective computing (AC), hypertension

## Abstract

Negative and positive emotions are the risk and protective factors for the cause and prognosis of hypertension. This study aimed to use five photoplethysmography (PPG) waveform indices and affective computing (AC) to discriminate the emotional states in patients with hypertension. Forty-three patients with essential hypertension were measured for blood pressure and PPG signals under baseline and four emotional conditions (neutral, anger, happiness, and sadness), and the PPG signals were transformed into the mean standard deviation of five PPG waveform indices. A support vector machine was used as a classifier. The performance of the classifier was verified by using resubstitution and six-fold cross-validation (CV) methods. Feature selectors, including full search and genetic algorithm (GA), were used to select effective feature combinations. Traditional statistical analyses only differentiated between the emotional states and baseline, whereas AC achieved 100% accuracy in distinguishing between the emotional states and baseline by using the resubstitution method. AC showed high accuracy rates when used with 10 waveform features in distinguishing the records into two, three, and four classes by applying a six-fold CV. The GA feature selector further boosted the accuracy to 78.97%, 74.22%, and 67.35% in two-, three-, and four-class differentiation, respectively. The proposed AC achieved high accuracy in categorizing PPG records into distinct emotional states with features extracted from only five waveform indices. The results demonstrated the effectiveness of the five indices and the proposed AC in patients with hypertension.

## 1. Introduction

A bidirectionality of emotion and disease was found in hypertension [1]. Specific emotions (e.g., anger, sadness, and depression) were linked to the psychopathological mechanisms and associated with the cause and prognosis of hypertension. For example, early studies focused on suppressed anger as a psychological risk factor for hypertension and carotid arterial stiffness in older adults [2], and later studies identified the association of anger-out and depressive symptoms with an increased risk of blood pressure (BP) progression after adjusting for other risk factors [3]. Studies reported that 4–37.1% of hypertension is comorbid with depressive symptoms [4,5]. A systematic review and meta-analysis of 41 studies revealed a 26.8% prevalence of depression in patients with hypertension, and the prevalence of depression determined through self-report questionnaires was slightly higher (29.8%) than through clinical interviews (21.3%) [6]. Studies reported that depressive symptoms were positively related to perceived stress, trait anger, state anger, and social conflict, and negatively related to ego resilience and health-promoting behaviors in patients with hypertension [5,7]. Positive emotions were associated with reduced BP after adjusting for relevant risk factors, and a one-point increase in positive emotion score on a four-item index of positive emotion from the Center for Epidemiologic Studies–Depression was associated with 3% and 9% decreased odds for BP in patients using and not using hypertension medication, respectively [8].

The results indicate that negative emotion is not only a risk factor for the development of hypertension but also increases the probability of cardiovascular disease and causes a poor prognosis among patients with hypertension. In contrast, numerous studies have confirmed that sustained and stable positive emotions lower blood pressure, lower physiological reactivity, accelerate cardiovascular recovery, stabilize body balance, and reduce the risk of incidents of hypertension and mortality in the long run [1,8,9]. However, emotional screening is not a routine procedure in clinical settings; moreover, several patients do not recognize their own emotions. Thus, evaluating negative emotions in patients with hypertension is the first step toward understanding the effect of diseases and preventing adverse prognoses. 

Owing to the rapid developments in digital signal processing and artificial intelligence (AI) in AC in recent years, several related technologies using physiological signals have been applied in studies on AC or emotional recognition [10]. Picard et al. [11] extracted temporal characteristics from facial electromyography (EMG), electrocardiogram (ECG), electrodermal activity (EDA), and respiration to identify eight different emotions (normal, anger, hate, grief, platonic love, romantic love, joy, and reverence). They recruited only one subject, achieving a recognition rate of 81.25%. Kim et al. [12] proposed the use of three physiological signals, namely ECG, EDA, and skin temperature, to identify four types of emotions (stress, anger, sadness, and surprise) with a support vector machine (SVM) classifier; the emotional recognition rate was 61.8% for 50 subjects. Wiem et al. [13] used four physiological signals, namely ECG, EDA, skin temperature, and respiration, to identify 20 types of emotions. They applied a feature selection approach to improve the effectiveness of the SVM classifier; the accuracy rate was 69.47% by using a combination of ECG and respiratory features. Udovičić et al. [14] calculated time- and frequency-domain features by using photoplethysmography (PPG) and EDA signals, and the accuracy rate was 67% by using an SVM classifier to differentiate two types of emotions (positive and negative). Pollreisz et al. [15] employed statistical features calculated based on the changes detected in the PPG, EDA, and skin temperature waveforms using a decision tree classifier to achieve 64.66% accuracy in differentiating four types of emotions (happiness, anger, sadness, and pain). Shahid et al. [16] proposed a fusion architecture to combine frequency transforms and statistical measures calculated based on ECG and PPG signals and tested them on nine classifiers. The accuracy in differentiating among four emotional states (sadness, disgust, fear, and happiness) ranged between 34% and 85.7%; among them, the ensemble bagging trees attained the highest accuracy. 

In the studies on affective computing, researchers attempted to extract adequate features from multimodal (multiple) physiological signals related to human emotions and applied the AC approach to different emotional states. However, in real-world applications, the use of multimodal signals complicates the system. Very few studies have focused on using a single signal for affective computing. PPG sensors, which are already utilized widely in wearable devices, are believed to be the most accessible and suitable for collecting profound emotion-relevant information. PPG has various advantages, such as easy implementation, portability, non-invasive collection of bio-signals, and detection of blood volume changes in the microvascular tissue bed or peripheral circulation [17]. The PPG shape is detected by illuminating the skin with infrared light from a light-emitting diode and capturing reflected light absorption in the skin of the fingertip, earlobe, or forehead. Each cardiac cycle appears as a pulse in the ECG, which can also be seen in the PPG as a distinct waveform. PPG signals include cardiac constriction and peripheral vessel pressure and are influenced by BP, the autonomic nervous system, and vascular compliance. During cardiac dilation, vascular pressure is reduced. Lin [18] pinpointed the relationship between the ECG and PPG waveform, where the systolic upstroke time (ST) indicates a direct pressure wave traveling from the left ventricle to the finger or ventricular rapid ejection time and left ventricular release of large blood. The PPG amplitude is influenced by cardiac output, ventricular ejection speed, arterial resistance, and blood-vessel-wall elasticity and reflects the blood volume change in the blood vessel underneath the PPG sensor [18]. The emotional and respiratory factors can lead to changes in waveform, frequency, and amplitude of PPG signals due to the neural regulation of the cardiovascular system on both macro- and microcirculatory levels [19].

Park et al. [20] tested the accuracy of identifying two types of emotions (happiness and sadness) using only the PPG signal and SVM classifier, and the accuracy rate was 63.67% for five subjects. Lee et al. [21] used a one-dimensional convolutional neural network (1D CNN) to extract PPG signal features for emotion classification; the emotion recognition accuracy was 75.3% in the valence (positive and negative) based on the Database for Emotion Analysis using Physiological Signals (DEAP database). Lu et al. [22] attempted to recognize the pulse of love at first sight based on PPG signals. A total of 26 features were calculated, and several classifiers were used for classification. The best accuracy achieved for the binary classification task before feature selection was 68.18%, which improved to 71.09% with the eXtreme Gradient Boosting (XGBoost) classifier after feature selection.

PPG captures not only the activities during the heart’s systolic and diastolic periods but also the hemodynamic, hemorheological, and network information of the peripheral microcirculation system [10,23]. Teng and Zhang [24] and Kurylyak et al. [25] defined the rising phase of the PPG waveform as ST or t1 and the falling phase as diastolic time (DT or t2). DT is the time duration between the diastolic peak of the pressure wave from the arteries of the lower body back to the finger [18,22]. Li [26] measured ST and DT values during happy and sad films (7 min) in 50 healthy participants and found longer DT and total time in the happiness feeling periods compared with the sadness feelings periods. However, there was no significant difference in ST between the happy and sad films. The researchers found that there was a lower blood volume amplitude (BVA) at the anger recall stage compared to the neutral recall stage and the baseline in patients with coronary artery disease [17,27]. 

The arterial wave propagation theory and PPG morphological theory have explored the relationship between PPG and BP [28]. Mitsutake et al. [29] performed a logistic regression analysis and found that a longer ST predicted a higher score in coronary artery calcification, which indicates a high risk of cardiovascular disease. Nakashima et al. [30] reported that ST was prolonged in patients with peripheral artery disease due to reduced blood flow when measured through angiography. Thus, the waveform of a pulse wave, such as ST, may be used as an index to diagnose the severity of coronary artery calcification, peripheral stiffness, or arterial narrowing. Teng and Zhang [24] recorded 18 s of ECG and PPG data during resting, 109-step climbing exercise, and recovery, and then analyzed four PPG characteristics. The results showed higher correlations among DT, systolic BP (SBP), and diastolic BP (DBP) compared to the width of the 1/2 pulse amplitude, width of the 2/3 pulse amplitude, and ST in 15 healthy subjects. Yoon [31] enrolled five healthy male adults and measured their BP and PPG during the resting baseline and a 100-step climbing exercise for five consecutive days. The results showed slightly higher correlations between ST and SBP than between DT and SPB (r (ST-SBP) = −0.6049 and r (DT-SBP) = −0.6046); moreover, the correlation between DT and DBP (r = −0.764) was higher compared with that between ST and DBP (r = −0.663). The DT showed a higher correlation with SBP and DBP compared with ST. Kurylyak et al. [25] examined the PPG and BP values and found that DT was negatively related to SBP and DBP. Similarly, Samria et al. [32] explored the relationships between PPG and BP and found a negative correlation between DT and DBP (r = −0.811) among 18–25-year-old healthy subjects, as well as a negative correlation between DT and SBP (r = −0.869) among 26–50-year-old healthy subjects. 

Considering PPG and BP in clinical populations, Kiuchi et al. [33] enrolled 3912 participants and divided them into peripheral artery disease (PAD) and non-PAD based on the ankle-brachial index (ABI) measurement. The results showed that patients with PAD had higher SBP, mean BP, pulse pressure, ST, and percentage of mean arterial pressure (%MAP) than patients without PAD. Shoji et al. [34] measured ABI and conducted invasive coronary angiography for patients with suspected coronary artery disease (CAD). The results showed that patients with CAD (at least one stenotic lesion > 50%) had higher ST than those without CAD, and ST was related to the severity of CAD, which was measured using the Gensini score and the synergy between PCI with Taxus and CABG (SYNTAX) score. 

However, previous studies have focused on healthy populations by applying AI technology in affective computing, whereas only a limited number of studies have been conducted on patients with hypertension. Moreover, ECG requires more measurement technology and pre-processing of physiological signals, whereas PPG involves non-invasive measurement and convenient analysis. Therefore, the aims of this study were based on Russel’s circumplex model of emotions to divide the dimensions of valence (negative/positive) and arousal (high/low), namely: (1) to conduct traditional statistical analysis and AI-enabled AC through PPG characteristics, including blood volume amplitude (BVA), ST, DT, peak-to-peak intervals (PPI), and valley-to-valley intervals (VVI) for different emotions in patients with hypertension; (2) to explore the correlations between ST, DT, and BP for different emotions in patients with hypertension; (3) to extract the amplitude and waveform features from the PPG signal recorded from patients with hypertension at different emotional stages. A powerful machine-learning classifier SVM was adopted to justify the capability of these features in differentiating distinct emotional states. Moreover, a feature selection approach was applied to determine the optimal feature combinations that achieved the best results.

## 2. Materials and Methods

### 2.1. Participants

**Figure 1:**
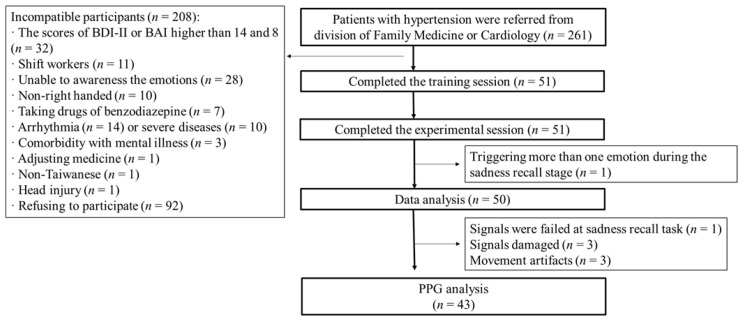
Participants’ study flow.

A total of 261 patients diagnosed with hypertension were referred by physicians at the divisions of Cardiology and Family Medicine of Kaohsiung Medicine University Hospital and Kaohsiung Municipal Siaogang Hospital. The inclusion criteria for hypertension were: (1) patients were stable and under prescription for at least three months; (2) according to the diagnosis criteria for hypertension (140/90 mmHg), patients with comorbid hyperlipidemia and overweight (body mass index ≥ 24) were included in this study; (3) age 30–70 years. The exclusion criteria were: (1) participants with arrhythmia, with a pacemaker, comorbid with severe physical illness (such as cancer, stroke, or heart failure), or mental disorders (such as major depressive disorder or substance use); (2) Beck Depression Inventory-II (BDI-II) and Beck Anxiety Inventory (BAI) scores were higher than 14 and 8, respectively; (3) patients with shift work, going through pregnancy, or under benzodiazepine medication. Fifty-one participants completed the pre-test; one patient’s PPG signals failed in the sadness recall task; three patients’ PPG signals were damaged; and three patients had movement artifacts. Finally, PPG data from 43 participants were included in the statistical analysis (Figure 1). 

The institutional review board of Kaohsiung Medical University Hospital approved this study. All participants provided written informed consent before the study. After completing all experimental procedures, participants received TWD 1000 (about USD 30). 

### 2.2. Materials

#### 2.2.1. Psychological Questionnaires

All participants completed self-report questionnaires, which included demographic characteristics (including age, sex, education, and marital status) and an emotional checklist and rating scale, the BDI-II and the BAI. The emotional checklist and rating scale were used to evaluate the specific emotion during emotional recall tasks, and the emotion rating was used to evaluate the emotional intensity (from 1 = not at all to 5 = very) in the past event and during the experimental stages. The 21-item BDI-II was used to measure the severity of depression. The 21-item BAI was used to measure anxiety severity. 

#### 2.2.2. Physiological Parameters

A non-invasive PPG sensor (BVP-Flex/Pro) was placed on the participant’s thumb and recorded continuously using ProComp Infiniti^TM^ version 6 (Thought Technology Ltd., Montreal, QC, Canada). The sensor generates 940 nm wavelength infrared light pulses against a skin surface and measures the amount of reflected light. The PPG signal was filtered with a preset 0.1–50 Hz bandpass filter and was acquired by the device at a sampling rate of 2048 samples/s. Patients’ SBP and DBP were measured at 3 min intervals using GE Marquette SmartPac Tram transport display (Absolute Medical Equipment, Garnerville, NY, USA). 

### 2.3. Experimental Procedure

**Figure 2:**
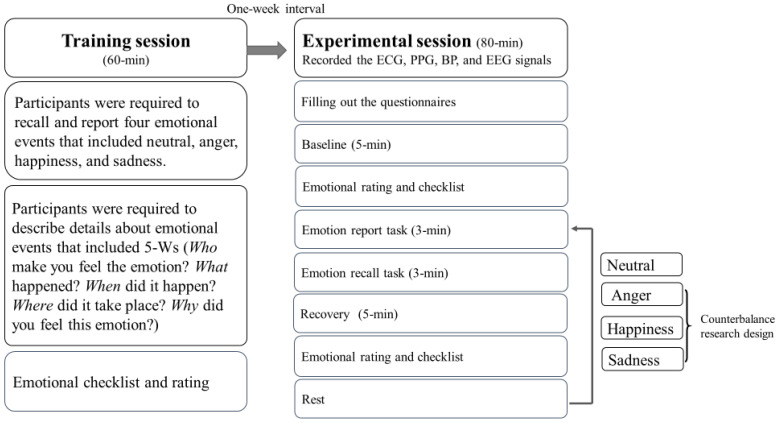
The procedure for the training and the experimental sessions, and the protocol for physiological signals measurement.

The participants were instructed to refrain from caffeinated beverages, alcohol, smoking, and excessive exercise 3 h before the experimental protocol. The participants were seated in a sound-attenuated and temperature-controlled room. Participants completed the demographic questionnaire and psychological questionnaires in the laboratory room and then participated in the training session. The training and experimental sessions were administered at one-week intervals, as per the following procedure (Figure 2). 

(1) Training session: Participants were required to recall and report four emotional states from their past life events, namely neutral, anger, happiness, and sadness. Participants were required to describe the emotional events in detail, which included answering the 5 Ws (“Who made you feel the emotion? What happened? When did it happen? Where did it take place? Why did you feel this emotion?”).

(2) Experimental session: After a one-week interval, participants were guided to a 5 min sitting baseline and then reporting and recalling the neutral event, followed by the other three emotional events (anger, happiness, and sadness), employing a counterbalance design for controlling the sequence effects. (1) The 6 min neutral recall task, including a 3 min report of a non-emotional neutral event that happened in the previous 6 months, e.g., “Please tell me what did you do yesterday”, was followed by a 3 min recall, wherein participants were seated comfortably; (2) the 6 min emotional recall task (the anger/sadness/happiness events were conducted by following a counterbalance design) included a 3 min report of an emotional event that occurred in the previous 6 months. For example, “Please tell me about an emotional event, including who made you feel the emotion? What happened? When did it happen? Where did it take place? Why did you feel this emotion?”, followed by a 3 min recovery. After each emotional report and recall, an emotional evaluation was conducted. PPG and BP were measured during the entire experimental session. After finishing the experimental session, the patients completed an emotional rating and checklist as a manipulation check.

### 2.4. Data Reduction and Statistical Analysis

This study focused on BP and PPG signals; the 3 min SBP and DBP data were acquired, and the 3 min PPG data were divided into six 30 s PPG indices of BVA, ST, DT, PPI, and VVI at baseline, neutral recall, anger recall, sadness recall, and happiness recall. In this study, we calculated the change score (Δ) to quantify the difference between the emotional state and baseline. For example, the change score of BVA (ΔBVA) at the anger stage indicated the anger recall of BVA minus the baseline BVA. 

Descriptive statistics of demographic data, psychological questionnaire scores, and physiological parameters were scored and analyzed using the Statistical Package for the Social Sciences version 21.0 (International Business Machines Corporation, Armonk, NY, USA). One-way repeated-measures analysis of variance (ANOVA) was used to examine the various experimental stage differences (baseline, neutral recall, anger recall, sadness recall, and happiness recall) on PPG and BP parameters. If the Mauchly sphericity test is satisfied, Bonferroni’s post hoc comparison will be applied; if the Mauchly sphericity test is violated, the Greenhouse–Geisser adjustment will be applied in one-way repeated-measures ANOVA. The effect size was calculated with partial eta-square (ηp^2^), where less than 0.06, 0.06–0.14, and more than 0.14 were considered small, medium, and larger effect sizes, respectively [35].

### 2.5. The Proposed AC Algorithm

**Figure 3:**
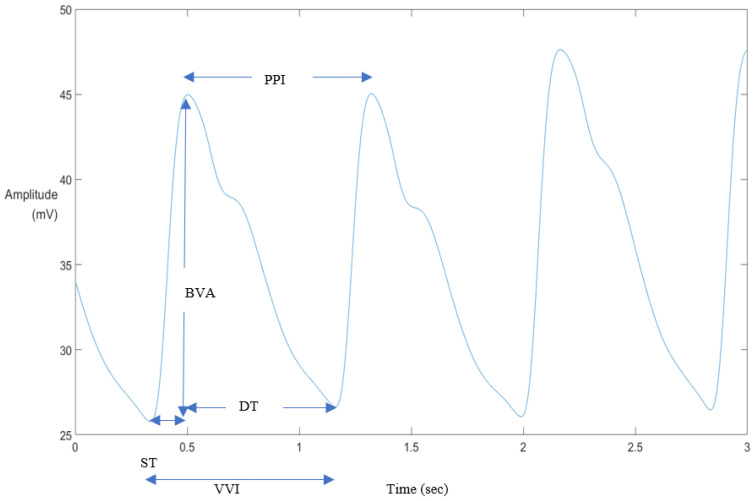
Significant waveform features extracted from the PPG signal.

(1) Feature extraction: The 3 min individual PPG signals acquired during the baseline and emotion recall stages were divided into six non-overlapping segments, each of which was 30 s in length. A typical PPG heartbeat cycle contains one peak bounded by two valleys, as shown in Figure 3. The peaks of the waveform can be detected by finding its local maxima, although we may need to set rules to exclude peaks at unreasonable distances from the previous ones. After the detection of all peaks, the valleys were identified by finding the minimum between consecutive peaks. As a result, we were able to measure the values of the five indices, namely BVA, ST, DT, VVI, and PPI. The BVA measures the height from the first valley to the peak. The ST and DT depict the time that elapses from the first valley to the peak and from the peak to the next valley, respectively. VVI and PPI represent the time distance between a pair of valleys and peaks, respectively. 

For each 30 s segment, the mean and standard deviation for all five indices were calculated as representative features, resulting in a total of 10 features. In summary, from each subject, we acquired six 30 s PPG segments recorded from the baseline and each of the four emotion recall states, and 10 features were calculated to characterize each 30 s segment. However, as the baseline properties of the subjects can be significantly different, we managed to tackle the problem of individual differences by using differential features. The idea is intuitive. The differential features were calculated by subtracting the baseline features from the activated state features, and their effects on differentiating distinct emotional states were evaluated. Moreover, the differentiating capability of the combined use of both activated state features and differential features was also justified.

(2) Feature normalization: Because the dynamic range of individual features can differ dramatically, a feature normalization process is usually required to scale all the features to the same level. In this study, we employed z-score normalization, where each feature was normalized by first subtracting the mean and then dividing by the standard deviation (STD). The mean and STD of each feature were calculated solely from the training dataset and used to normalize that feature in both the training and testing datasets. 

(3) Feature selection: In a classification task, when the feature dimensions are high, feature selection techniques are usually required to determine the most representative subset of features, which can efficiently delineate the primary feature set and remove redundant features, such that the dimensions are reduced, while the accuracy is retained or even improved. For a feature set containing n features, the possible combinations of feature subsets are (2^n^ − 1). In this study, we compared the differentiating power of 10 original features, 10 differential features, and a combination of both original and differential features (20 features). We applied the full search approach to a smaller feature set with 10 features. However, for the feature set with 20 features, we exploited a (almost) global optimal approach, the genetic algorithm (GA), to reduce the computational load and accelerate the selection process.

GA is an optimization methodology based on Darwinian evolution theory [36] and was first introduced in the literature by Holland [37]. Basic arithmetic algorithms have been proposed for typical evolutionary operations, such as selection, crossover, and mutation. The genes are modeled by binary strings called chromosomes. To use GA for feature selection, we associate the binary representation of a chromosome with a specific combination of features, such that a “1” represents selection, whereas a “0” represents the removal of a specific feature from the feature set.

The selection process uses classification accuracy as the fitness function. Chromosomes with high fitness values are selected as parents at a higher probability. The crossover process produces new chromosomes, with a certain possibility of mutation in the next generation. In this manner, the GA generates and modifies chromosomes until a preset number of generations is reached or the optimal fitness values remain constant. Because the GA operates on a collection of candidate solutions in parallel, and the evolution rules allow the algorithm to jump out of local optima, the GA has a higher probability of finding the global optimal solution. As a result, the GA generates an optimal combination of features that possess the highest discrimination power.

(4) Classifier: An SVM [38] was employed as the classifier in this study. The SVM maps the training samples from the input space into a higher dimensional feature space using a kernel function. Any product of the vectors in the optimization process can be implicitly computed to generate a hyperplane to categorize two classes. When the training data are not completely separable, the optimal solution can be found by minimizing both the empirical risk and complexity of the hypothesis space. Multiple SVM classifiers can be integrated using the one-against-one or one-against-all approach to treat problems with more than two classes. In this study, we used the one-against-all approach [39] to differentiate three and four categories of emotions. A radial basis function (RBF) kernel was used in this study.

(5) Validation: We employed two methods to validate the capabilities of the features and classifiers in emotion recognition from different points of view. (a) Resubstitution validation: This method is also called the all-train-all-test (ATAT) or self-consistency evaluation. The purpose of this validation is to test the differentiating power of the proposed method by categorizing the entire database in a classifier model construction process. (b) K-fold cross-validation: This method tests the capability of the features and classifiers in identifying similar data, given that information from the same group of subjects is provided. The data across subjects and emotional states were divided evenly into K folds. As we divided the signal recordings from individual experimental settings into six 30 s segments, we applied six-fold cross-validation (CV). Each fold of features had the same chance to serve as testing data, and the other five folds were used to train the classifier. The accuracies of the six individual trials were averaged to verify the performance of the classifier across the dataset.

## 3. Results

### 3.1. Participants’ Characteristics

The demographic data are shown in Table 1. Forty-three patients completed the statistical analysis (34.88% females and 65.12% males). The emotional checklist showed the main effects on anger, happiness, and sadness (anger: F = 1186.77, *p* < 0.001, ηp^2^ = 0.97; happiness: F = 2282.92, *p* < 0.001, ηp^2^ = 0.98; sadness: F = 1836.24, *p* < 0.001, ηp^2^ = 0.98). These results indicate that the experimental procedure can induce the patients’ emotions of anger, happiness, and sadness (Table S1). Moreover, the patients reported an emotional intensity of 97.69%, 95.49%, and 96.30% for anger, happiness, and sadness, respectively, in the experimental session compared with their past experiences. 

### 3.2. Physiological Responses under Different Experimental Stages

Regarding BP, the one-way repeated-measures ANOVA revealed a significant interaction effect on SBP and DBP (F = 26.32, *p* < 0.001, ηp^2^ = 0.39; and F = 39.07, *p* < 0.001, ηp^2^ = 0.48, respectively), and the Bonferroni post hoc comparison showed higher SBP and DBP at the anger, happiness, and sadness stages compared with baseline and neutral stages. Regarding PPG parameters, the one-way repeated-measures ANOVA revealed a significant interaction effect on BVA, ST, and DT (F = 21.01, *p* < 0.001, ηp^2^ = 0.33; F = 15.36, *p* < 0.001, ηp^2^ = 0.27; and F = 5.90, *p* = 0.001, ηp^2^ = 0.12, respectively). The Bonferroni post hoc comparison showed a lower BVA at anger, sadness, and happiness recall stages compared with that at baseline and neutral recall stages, as well as higher ST at neutral, anger, sadness, and happiness recall stages compared with that at baseline, and higher ST at the anger recall stage compared with that at the neutral recall stage. Moreover, the DT was lower at the anger and sadness recall stages than that at the baseline. However, there were no significant differences in PPI and VVI at the neutral, anger, sadness, and happiness recall stages (F = 2.19, *p* = 0.073, ηp^2^ = 0.05; F = 1.39, *p* = 0.252, ηp^2^ = 0.03, respectively; Table 2). 

This study analyzed changes in BP and PPG parameters to differentiate the four emotional states. The results showed higher SBP and DBP values at the anger, happiness, and sadness stages compared with those at the neutral stage (F = 11.59, *p* < 0.001, ηp^2^ = 0.22; and F = 17.08, *p* < 0.001, ηp^2^ = 0.29, respectively), as well as lower BVA at the anger, happiness, and sadness stages compared with that at the neutral stage (F = 16.17, *p* < 0.001, ηp^2^ = 0.28). Moreover, ST was higher at the anger and sadness stages compared with that at the neutral stage (F = 7.31, *p* = 0.001, ηp^2^ = 0.15), and DT was lower at the sadness stage compared with that at the neutral stage (F = 3.97, *p* = 0.016, ηp^2^ = 0.09). However, there were no significant differences in PPI and VVI at the neutral, anger, sadness, and happiness stages (F = 2.89, *p* = 0.051, ηp^2^ = 0.06; F = 1.76, *p* = 0.167, ηp^2^ = 0.04) (Table 2). 

### 3.3. Correlations between PPG Parameters and BP

The results demonstrated a negative correlation between DT and DBP (r = −0.37~−0.53) and among PPI, VVI, and DBP (r = −0.35~−0.46) at the baseline, neutral, anger, happiness, and sadness recall stages. Moreover, we also found a negative correlation between BVA and DBP at the sadness recall stage (r = −0.40, *p* < 0.01) and a negative correlation between ST and DBP at the baseline (r = −0.36, *p* < 0.05) (Table 3).

### 3.4. AC through PPG parameters

(1) Differentiation between the baseline and emotionally activated stage: The AC algorithm was first applied to different individual emotionally activated stages from the baseline (Table 4). By applying resubstitution validation, the proposed AC algorithm achieved 100% accuracy in differentiating all emotionally activated stages from the baseline. This result supports the feasibility of using the AC algorithm to construct an effective model for completely differentiating the emotionally activated stages from the baseline for an entire dataset. When we applied the six-fold CV method to justify the differentiating power of the AC algorithm by providing only 5/6 of the data while testing with the other 1/6, high accuracy (more than 85%) could also be observed in differentiating the anger, happiness, and sadness stages from the baseline (85.47%, 86.24%, and 87.40%, respectively). The differentiating power of neutral from baseline was 71.12% (Table 4). 

(2) Differentiation among distinct emotionally activated stages: The capability of the AC algorithm to differentiate distinct emotional records was tested by observing its accuracy in categorizing the records into two (positive (neutral and happiness) and negative (anger and sadness)), three (negative (anger and sadness), neutral, and positive (happiness)), and four (anger, sadness, neutral, and happiness) classes. In the two-class (2C) categorization, the negative class included anger and sadness states, and the positive class included neutral and happiness states. In the three-class (3C) categorization, the neutral state was separated from the positive class as a distinct class. The four-class (4C) categorization included four emotional states. We tested the differentiating power of the AC algorithm using different feature sets and validation methods. The capabilities of the AC algorithm in the emotional state categorization for the entire dataset were justified using the resubstitution (all-train-all-test, ATAT) validation method. Notably, by using the resubstitution (ATAT) validation, the proposed AC algorithm achieved 100% accuracy in all the emotional state categorization tests. This result demonstrates the powerful capability of the AC algorithm in categorizing the emotional states for the entire dataset. 

The capabilities of the AC algorithm in emotional state categorization across data segments were justified using the six-fold CV method (Table 5). The predictability of the AC algorithm in emotion categorization across data segments (using six-fold CV) is impressive. The accuracy is relatively high when compared to other emotional state identification studies (please refer to the Discussion section). The accuracy is the highest with 20 combined features, and the accuracy after using 10 differential features outperforms that of using 10 waveform features. However, the trends in emotional type categorization were similar. The record categorization accuracy decreased in the order 2C, 3C, and 4C, which is not surprising because the classification tasks become tougher with the increasing number of classes. These observations highlight the importance of using differential features to characterize the changes in individual features between the activated and baseline states. Moreover, the combined use of both waveform and differential features further improves the performance. 

The effects of feature selection in AC for emotional type categorization are also included in Table 6 for comparison. It is obvious that using feature selectors further improves the accuracy of certain categorization tasks, although the accuracy order remains the same, that is, 2C, 3C, and 4C. The effect of using feature selectors on 20 combined features is the most promising; a 3.39–5.72% improvement in the accuracy is observed for different categorization tasks. The accuracy of a few AC algorithms also increased after applying the feature selectors separately to the 10 differential and 10 waveform features, respectively, although to a minor extent. Similar to the tests without feature selectors, the differential feature case outperformed the waveform feature case.

## 4. Discussion

The quality of the PPG signal is a major factor, which influences the reliability of waveform features. The pre-processing methods, especially filtering, can significantly change the PPG waveform features. In this study, we acquired the PPG signal using an FDA-approved system with an adequate sampling rate and bandpass filter, which enabled preserving reliable PPG morphological features for further analysis.

Regarding the correlations between ST, DT, and BP, the results demonstrated negative correlations between DT and DBP for different emotional states in patients with hypertension. This result was consistent with prior studies, which reported similar observations [24,25,32]. Moreover, negative correlation between BVA and DBP under the sadness emotion and lower BVA under the sadness emotion, indicating vasoconstriction, may cause higher DBP. This study found that DBP values under positive emotion (happiness) and negative emotion (anger and sadness) were negatively related to DT, PPI, and VVI. This result indicated that longer diastolic time and interbeat intervals (peak-to-peak intervals and valley-to-valley intervals) were related to lower DBP; the underlying physiological mechanisms may be related to lower reactivity under both positive and negative emotions [9,40]. Therefore, different emotions cannot be distinguished by using traditional statistical methods. Several studies used PPG to predict hypertension; these studies converted the PPG features to pulse arrival time (PAT), PPG amplitude, PPG waveform area, and slope [41,42,43]. Lan et al. [41] used PPG-derived HRV signals to discriminate between participants with or without hypertension; they found six HRV parameters to predict hypertension, and the SDNN of HRV had the highest accuracy of 85.47% for predicting hypertension. Liang et al. [42] found that combining the PAT and PPG features can reach an accuracy of 88.49%.

The proposed AC algorithm displayed high accuracy in differentiating the anger, happiness, and sadness stages from the baseline. Slightly lower, yet noticeable, accuracy was attained in differentiating the neutral stage from the baseline. These observations demonstrate the importance of using features calculated from the five waveform indices in emotion differentiation, which had higher accuracy rates than traditional statistical analysis using repeated-measures ANOVA. The ANOVA differentiated between neutral and baseline using SBP, DBP, BVA, and ST; between anger and neutral using ST; and between sadness, happiness, and baseline using DT. However, it could not distinguish between anger, happiness, and sadness. Moreover, the higher accuracy achieved using the 10 differential features when compared with that using the 10 waveform features highlights the importance of using differential features in emotional state categorization. The benefit of using differential features can also be observed in traditional statistical analysis using repeated-measures ANOVA.

The selected items from 20 combined features using GA in the 2C, 3C, and 4C categorization tasks are summarized in Table 6 (denoted as cross “X” symbols). For each of the five indices, four features were calculated, including the mean, STD, differential mean, and differential STD. The frequency of a feature being selected demonstrates the relative significance of the feature and the associated indices in emotional state identification. The results show that all four features associated with BVA, the mean and differential mean of ST, the mean and differential mean of DT, the mean of PPI, and the mean, STD, and differential mean of VVI contributed profoundly to all three emotion identification tasks. Among the five indices, the BVA features were selected most frequently, and thus, BVA was inferred to be the most crucial index in emotional categorization. Among the four features for each of the indices, the “mean” was selected by all the classification tasks, followed by the “differential mean” associated with BVA, ST, DT, and VVI. The “STD” feature of BVA and VVI and the “differential STD” feature of BVA were also demonstrated to be significant in the classification tasks. Moreover, the differential features were selected at similar frequencies when compared to their original waveform counterparts, confirming their significance in emotional state differentiation; however, the two categories of features needed to complement each other to fulfill the classification tasks. Although we can identify several substantial features in the study, it is noteworthy that other less dominant features must be recruited to supplement the classification capability of these features.

Khalid et al. [44] selected the 3 most significant PPG pulse features (total area, rising time, and width 25%) out of 16 time-based signal features, based on a statistical multicollinearity test and a two-step method, for blood pressure estimation. Although they used normalized amplitude and normalized time (w.r.t. VVI) for extracting the features, the three significant PPG pulse features are closely related to the five morphological features proposed in this study for emotional state recognition. More specifically, the (normalized) total area is related to both BVA and VVI, the (percentage of) rising time to ST and DT, and the width 25% to ST, DT, VVI, and PPI. This consistency may imply the close relationship between blood pressure and emotional states. However, our study employs the raw features instead of the normalized ones, which may provide more profound information for strengthening the distinguishability of the proposed morphological features in emotion recognition. The addition of differential features, which measure the change of features between the baseline and the activated states, further boosts the distinguishability of the proposed AC (Table 5).

The performance of the proposed AC algorithm was compared with that of four representative methods in the literature. The results are presented in Table 7. A summary of these methods is provided in the Introduction. Among them, the methods proposed by Park et al. [20], Lee et al. [21], and Lu et al. [22] used only PPG signals to differentiate two types of emotions. In contrast, Pollreisz et al. [15] used three types of signals (PPG, EDA, and skin temperature) to differentiate four types of emotions. This is because very few studies, if any, attempted to differentiate more than two types of emotions using only PPG. Notably, the proposed AC algorithm selected features originating from only five PPG waveform indices and achieved 78.97%, 74.22%, and 67.35% accuracy rates in categorizing the emotional states into two, three, and four classes, respectively. Compared with other methods, the proposed AC algorithm outperforms them in differentiating two categories of emotions and is competitive in differentiating four categories of emotions. Moreover, the proposed AC algorithm extracted features from only five indices that were relatively easy to measure and were tested on a relatively large population (number of subjects), which was more likely to result in lower accuracy in a user-independent setting. Although the experimental arrangement and validation methods may vary widely across different studies, the results demonstrate the significance of the five indices and the effectiveness of using the proposed AC algorithm in emotion recognition.

This study has a few limitations. First, due to the strict screening of hypertension patients, only 43 patients were included in AI AC, and the small sample size is a limitation of this study. Second, we evaluated only four types of emotions and used PPG signals to develop AI-assisted AC. The breadth of emotions may not cover all emotional levels of valence and arousal in Russel’s circumplex model of emotions. Third, this study only recruited patients with hypertension for affective computing and did not compare the PPG features with healthy controls. The psychophysiological mechanism of emotionally induced changes between patients with hypertension and healthy controls could be different and needs more future studies.

## 5. Conclusions

In summary, the proposed AI-assisted AC achieved high accuracy in categorizing four emotional states through five waveform indices of extracted PPG features. The results demonstrated the effectiveness of AI in AC in discriminating between neutral emotion, anger, happiness, and sadness in patients with hypertension. Future research can cover more emotion categories for employing AI in AC.

## Figures and Tables

**Table 1:** The demographic data, psychological questionnaires, and blood pressure (n = 43).

Variables		Mean (SD) or n (%)
Age, years		53.84 (8.87)
Sex, n (%)	Female	15 (34.88%)
	Male	28 (65.12%)
Education, n (%)	Primary school	1 (2.33%)
	Junior high school	3 (6.98%)
	Senior/vocational high school	13 (30.23%)
	Junior college	10 (23.26%)
	Bachelor degree	11 (25.58%)
	Master degree	5 (11.63%)
Marital status, n (%)	Single	4 (9.30%)
	Married	29 (67.44%)
	Divorced/separated	6 (13.95%)
	Widowed	4 (9.30%)
Beck Depression Inventory-II	Total score	3.16 (3.24)
Beck Anxiety Inventory	Total score	1.91 (2.03)
Systolic blood pressure (mmHg)		130.23 (11.31)
Diastolic blood pressure (mmHg)		80.33 (10.03)

**Table 2:** The raw and change scores of blood pressure and photoplethysmography at different experimental stages (n = 43).

	Baseline	Neutral Recall	Anger Recall	Happiness Recall	Sadness Recall	F	*p*	ηp^2^	Bonferroni Post hoc Comparison
	(1)	(2)	(3)	(4)	(5)				
SBP (mmHg)	130.23 (11.31)	135.79 (12.43)	140.51 (12.74)	140.98 (13.95)	144.77 (17.69)	26.32 ***	<0.001	0.39	3,4,5 > 1,2; 2 > 1
DBP (mmHg)	80.33 (10.03)	83.95 (9.98)	87.77 (10.32)	88.19 (10.94)	90.33 (11.02)	39.07 ***	<0.001	0.48	3,4,5 > 1,2; 2 > 1
BVA (mV)	11.82 (4.22)	11.13 (4.25)	8.23 (2.89)	8.89 (3.13)	8.39 (4.55)	21.01 ***	<0.001	0.33	3,4,5 < 1,2
ST (ms)	196.01 (50.12)	205.59 (57.01)	226.18 (40.75)	218.83 (41.22)	219.45 (41.08)	15.36 ***	<0.001	0.27	2,3,4,5 > 1; 3 > 2
DT (ms)	650.84 (82.39)	642.18 (84.08)	627.04 (61.02)	635.92 (79.94)	618.28 (101.20)	5.90 **	0.001	0.12	3,5 < 1
PPI (ms)	846.62 (113.40)	847.44 (117.47)	853.21 (114.07)	854.92 (117.02)	836.68 (116.75)	2.19	0.073	0.05	
VVI (ms)	846.90 (113.47)	847.91 (117.20)	853.36 (81.51)	854.95 (99.44)	838.11 (116.82)	1.39	0.252	0.03	
ΔSBP (mmHg)	-	5.56 (6.40)	10.28 (9.35)	10.74 (8.74)	14.53 (14.65)	11.59 ***	<0.001	0.22	3,4,5 > 2
ΔDBP (mmHg)	-	3.63 (3.39)	7.44 (5.94)	7.86 (5.08)	10.00 (7.84)	17.08 ***	<0.001	0.29	3,4,5 > 2
ΔBVA (mV)	-	−0.69 (3.16)	−3.59 (3.62)	−2.93 (3.92)	−3.43 (4.15)	16.17 ***	<0.001	0.28	3,4,5 < 2
ΔST (ms)	-	9.58 (16.50)	30.17 (33.24)	22.83 (28.52)	23.44 (29.36)	7.31 **	0.001	0.15	3,5 > 2
ΔDT (ms)	-	−8.67 (27.28)	−23.79 (50.57)	−14.92 (48.24)	−32.56 (61.29)	3.97 *	0.016	0.09	5 < 2
ΔPPI (ms)	-	0.81 (22.29)	6.58 (42.19)	8.29 (45.72)	−9.94 (60.79)	2.89	0.051	0.06	
ΔVVI (ms)	-	1.01 (22.21)	6.47 (60.64)	8.05 (49.36)	−8.79 (60.47)	1.76	0.167	0.04	

* *p* < 0.05, ** *p* < 0.01, *** *p* < 0.001. Note: Δ change score = emotional state–baseline; 1 = baseline; 2 = neutral recall; 3 = anger recall; 4 = happiness recall; 5 = sadness recall; BVA = blood volume amplitude; ST = systolic upstroke time; DT = diastolic time; STD = standard deviation; PPI = peak-to-peak intervals; VVI = valley-to-valley intervals.

**Table 3:** The correlations between photoplethysmography parameters and blood pressure under different experimental stages (n = 43).

	Baseline		Neutral Recall		Anger Recall		Happiness Recall		Sadness Recall	
	SBP	DBP	SBP	DBP	SBP	DBP	SBP	DBP	SBP	DBP
BVA (mV)	0.19	0.09	−0.08	−0.01	−0.08	−0.07	−0.23	−0.21	−0.27	−0.40 **
ST (ms)	−0.09	−0.36 *	−0.11	−0.24	0.16	−0.03	0.02	−0.03	0.13	0.02
DT (ms)	0.09	−0.37 *	0.01	−0.41 **	−0.10	−0.37 *	−0.09	−0.41 **	−0.26	−0.53 **
PPI (ms)	0.03	−0.43 *	−0.05	−0.41 **	−0.07	−0.45 **	−0.09	−0.38 *	−0.19	−0.46 **
VVI (ms)	0.03	−0.43 *	-0.05	−0.41 **	0.004	−0.30	−0.07	−0.35 *	−0.18	−0.45 **

* *p* < 0.05, ** *p* < 0.01 Note: SBP = systolic blood pressure; DBP = diastolic blood pressure; BVA = blood volume amplitude; ST = systolic upstroke time; DT = diastolic time; STD = standard deviation; PPI = peak-to-peak intervals; VVI = valley-to-valley intervals.

**Table 4:** Performance of affective computing for emotion activation.

Validation	Neutral	Anger	Happiness	Sadness
Resubstitution (ATAT)	100%	100%	100%	100%
Six-fold	71.12%	85.47%	86.24%	87.40%

Note: ATAT = all-train-all-test.

**Table 5:** Performance of affective computing in emotional type categorization using six-fold cross-validation method.

Feature Selection	Feature Types	Two-Class	Three-Class	Four-Class
	10 waveform features	68.90%	61.24%	52.91%
N/A	10 differential features	70.83%	66.47%	54.17%
	20 combined features	74.61%	70.83%	61.63%
	10 waveform features	68.90%	61.24%	53.00%
Yes	10 differential features	71.03%	66.47%	54.36%
	20 combined features	78.97%	74.22%	67.35%

Note: N/A = not available.

**Table 6:** Features selected from the 20 combined features using GA.

	BVA			ST			DT			PPI			VVI		
	2C	3C	4C	2C	3C	4C	2C	3C	4C	2C	3C	4C	2C	3C	4C
Mean	X	X	X	X	X	X	X	X	X	X	X	X	X	X	X
STD	X	X	X			X							X	X	X
Differential mean	X	X	X	X	X	X	X	X	X			X	X	X	X
Differential STD	X	X	X		X	X		X		X	X				

Note: BVA = blood volume amplitude; ST = systolic upstroke time; DT = diastolic time; STD = standard deviation; PPI = peak-to-peak intervals; VVI = valley-to-valley intervals; 2C = two classes of emotion types (happiness and sadness); 3C = three classes of emotion types (neutral, positive (happiness) and negative (anger, sadness)); 4 C = four classes of emotion types (neutral, anger, happiness, and sadness).

**Table 7:** Comparison with other relevant methods.

Method	Signal Types	Emotion Types	Features	Accuracy	Subjects
Park et al. [19]	PPG	2 (happiness and sadness)	1	63.67%	5
Lee et al. [20]	PPG	2 (positive and negative)	By 1D CNN	75.3%	32
Lu et al. [21]	PPG	2 (neutral and love)	26	71.09%	46
Pollreisz et al. [15]	PPG + EDA + skin temperature	4 (happiness, anger, sadness, and pain)	4	64.66%	16
This study	PPG	2 (positive (happiness) and negative (anger and sadness))	13	78.97%	43
	PPG	3 (neutral, positive (happiness) and negative (anger and sadness)	15	74.22%	43
	PPG	4 (anger, sadness, neutral, and happiness)	15	67.35%	43

Note: 1D CNN = one-dimensional convolutional neural network; EDA = electrodermal activity; PPG = photoplethysmography.

## Data Availability

Data can be obtained by contacting the corresponding author.

## Supplementary Materials

The following supporting information can be downloaded at: https://www.mdpi.com/article/10.3390/s22228771/s1, Table S1: The emotional checklist and rating.
