# Supplementary material for: Affective Computing Based on Morphological Features of Photoplethysmography for Patients with Hypertension
Source: Sensors (Basel). 2022 Nov 13;22(22):8771. doi: 10.3390/s22228771 (PMC9698908; doi:10.3390/s22228771)
Supplement: Supplementary file 1 [file sensors-22-08771-s001.zip › sensors-1963766-supplementary.pdf]

Supplement Table S1. The emotional checklist and rating.

Emotion checklist	Emotion	Anger (1) Mean (SD)	Happiness (2) Mean (SD)	Sadness (3) Mean (SD)	F	p	ηp2	Bonferroni Post hoc comparison
	Anger	4.37 (0.58)	1.00 (0.00)	1.07 (0.26)	1186.77***	<0.001	0.97	1 > 2,3
	Happiness	1.00 (0.00)	4.60 (0.49)	1.00 (0.00)	2282.92***	<0.001	0.98	2 > 1,3
	Sadness	1.02 (0.15)	1.00 (0.00)	4.53 (0.50)	1836.24***	<0.001	0.98	3 > 1,2
Emotion rating (%)		Past event	During experimental	Percentage of emotion was induced (%)				
Anger		88.14 (10.91)	85.23 (12.34)	97.69 (16.11)				
Happiness		94.30 (6.95)	89.77 (8.31)	95.49 (9.03)				
Sadness		91.98 (8.87)	95.49 (9.03)	96.30 (11.70)				

*p < 0.05, **p < 0.01, ***p < 0.001
